# Multispectral near infrared absorption imaging for histology of skin cancer

**DOI:** 10.1002/jbio.201960080

**Published:** 2019-11-05

**Authors:** Alexander Spreinat, Gabriele Selvaggio, Luise Erpenbeck, Sebastian Kruss

**Affiliations:** ^1^ Institute of Physical Chemistry Göttingen University Göttingen Germany; ^2^ Department of Dermatology, Venereology and Allergology University Medical Center Göttingen Germany

**Keywords:** diagnostics, histology, multispectral imaging, near infrared spectroscopy, skin cancer

## Abstract

Multispectral imaging combines the spectral resolution of spectroscopy with the spatial resolution of imaging and is therefore very useful for biomedical applications. Currently, histological diagnostics use mainly stainings with standard dyes (eg, hematoxylin + eosin) to identify tumors. This method is not applicable in vivo and provides low amounts of chemical information. Biomolecules absorb near infrared light (NIR, 800‐1700 nm) at different wavelengths, which could be used to fingerprint tissue. Here, we built a NIR multispectral absorption imaging setup to study skin tissue samples. NIR light (900‐1500 nm) was used for homogenous wide‐field transmission illumination and detected by a cooled InGaAs camera. In this setup, images I(*x*, *y*, *λ*) from dermatological samples (melanoma, nodular basal‐cell carcinoma, squamous‐cell carcinoma) were acquired to distinguish healthy from diseased tissue regions. In summary, we show the potential of multispectral NIR imaging for cancer diagnostics.

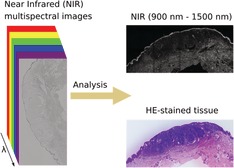

## Graphical abstract

Biomolecules absorb light in the near infrared region of the spectrum. Multispectral near infrared imaging of tissue samples allows to distinguish tumor from nontumor tissue.

## 1 INTRODUCTION

The diagnosis of cancer and the decision whether a cancer has been removed completely is a very important task in modern medicine. Up to now, different approaches and techniques have been developed and used. One tool is staining with hematoxylin and eosin (HE staining), which has been established and employed for more than 100 years in histology 1. This technique has been the benchmark for histological diagnostics for decades and can still be considered the gold standard today 2. However, staining techniques in general require thorough processing of the tissue, including fixation, embedding and sectioning and can therefore only be applied in excised tissue, preventing its use in vivo. Especially in dermatology, noninvasive methods could be very beneficial to determine, how far a tumor has already penetrated into the surrounding and underlying tissue and how extensive an excision must be for the patient to benefit from it. Procedures that provide in vivo diagnostics are rare and they have certain drawbacks concerning their ease of application and their accuracy 3. Currently existing, noninvasive techniques that are able to approximately discriminate between tumors (ie, benign and malignant skin), as discussed below, require great experimental efforts 4. Therefore, there is a high demand for simple, cost effective and accurate methods for diagnostics.

In the past, spectroscopy was explored to distinguish between tissue samples including skin samples 2. The spectroscopic investigation of in vivo samples was performed in different setups and it showed that it is in general possible to distinguish between different diagnoses also in the near infrared (NIR) region 5, 6. Noninvasive tools are also available in the dermatological field. Very common methods are confocal laser scanning microscopy (CLSM), Raman spectroscopy or multispectral imaging (in the visible range) 3, 4, 7, 8. In the case of CLSM, the setup requires a laser and a complex microscopic setup. Additionally, the penetration depth of this method is usually only about 200 μm 3, nevertheless, it was already shown in the past that CLSM can discriminate benign nevi from melanoma in vivo 9. Optical coherence tomography (OCT) is a popular technique that, although not yet a routine technique, is being used in several specialized dermatological practices and hospitals. OCT penetrates the skin up to 1.5 mm and it has been mainly used for diagnostics of basal‐cell carcinoma 3.

However, these noninvasive diagnostic methods have certain disadvantages with regards to specificity, penetration depth, speed or cost. Multispectral imaging is a method that is used in different fields and applications. It combines the spectral resolution of spectroscopic methods with the spatial resolution of imaging methods. In the past, it was typically used for spoilage detection in food industry 10, evaluation of agricultural products 11 and medical diagnostics 12. For medical diagnostics, not only the spectral information but also the spatial information is crucial to understand how a tumor is distributed within the tissue. For medical applications, the NIR range is interesting because this light penetrates biological tissues for depths up to about a few millimeters 13. Therefore, NIR methods have attracted a lot of interest, for example, the detection of biomolecules such as neurotransmitters, proteins, lipids and sugars with NIR fluorescent nanomaterials 14, 15, 16, 17, 18, 19, 20, 21, 22. Additionally, such NIR fluorescent nanomaterials have been used to label cell surface receptors, and for microrheology in tissue slices as well as in vivo 23, 24, 25. Biological samples furthermore absorb light in the NIR range to a certain extent, depending on their chemical composition 10. Therefore, multispectral imaging in this spectral range offers the possibility to be used as a noninvasive diagnostic tool in the field of dermatology or even for image‐guided surgery.

**Figure 1:**
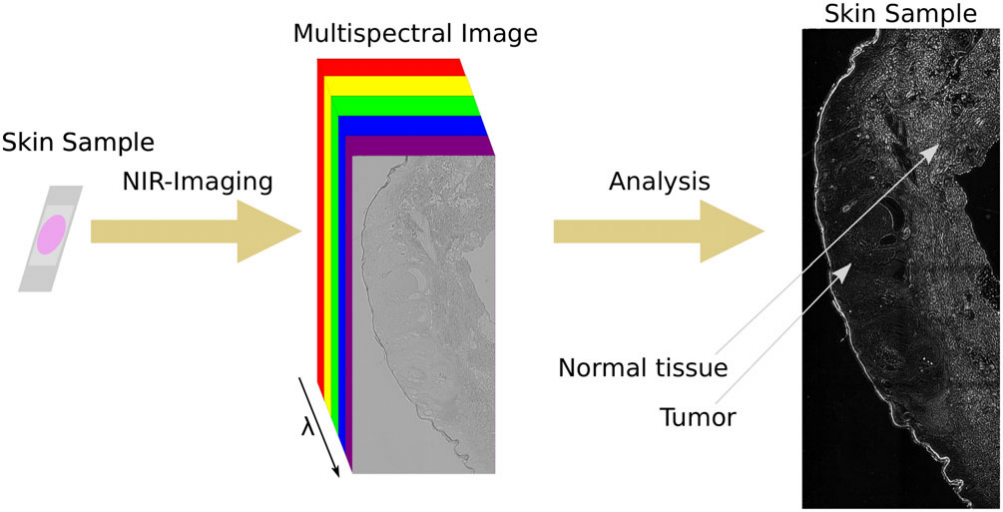
Concept of multispectral near infrared (NIR) absorption imaging of tissue samples. A histological sample is imaged with a multispectral imaging setup in the NIR spectral range and a multispectral image is so generated. Next, a simple mathematical analysis is carried out in order to exploit the so‐obtained spectral information for the discrimination between tumor and nontumor tissue by contrast

In this study, we develop a NIR multispectral imaging concept and use it to investigate fixed skin samples (Figure 1). We investigate the potential for tumor discrimination in histological samples of melanoma, nodular basal‐cell carcinoma and squamous‐cell carcinoma in the spectral range from 900 to 1500 nm.

## 2 MATERIALS AND METHODS

### 2.1 NIR multispectral absorption imaging

The setup consisted of a monochromator (MSH‐150, LOT‐Quantum Design, GmbH, Germany, f/4.6, focal length = 150 mm) equipped with a xenon‐arc lamp (300 W) and a diffraction grating (MSG‐T‐1200‐500, 30 mm × 30 mm, 1200 grooves/mm) together with an Olympus IX73 inverted microscope (Olympus, Japan) equipped with ×20, ×10 (Olympus UPlan FL N, ×10/0.30) and ×5 (Olympus MPlan N, ×5/0.10) objectives. The monochromatic light was directed to a Xenics Xeva‐1.7‐320 NIR camera (Xenics, Belgium). The microscope was equipped with an automated stage (ZABER ASR100B120B‐T3, Canada) that was used to acquire the images in a defined and controlled manner.

The data acquisition was carried out with the software from the camera manufacturer (*Xeneth64)*. The camera was calibrated before every measurement, and the change of wavelength of the illuminating light was performed by the monochromator manufacturer software *LOT‐Monochromator‐Suite*. In Xeneth64, the snapshot modus was used while the LOT‐Monochromator‐Suite scanned through wavelengths between 900 and 1500 nm automatically in steps of 20 nm after 2.5 seconds. The exposure time was set to 80 ms. The grid was defined by hand and the positions were actuated with the stage software *Zaber*. The initial grid position was defined, and the images were acquired at all defined wavelengths. Subsequently, the stage was moved to the next position and the next set of images was acquired.

### 2.2 Stitching

Large‐scale images were put together using the software *Fiji* 26. By defining the image positions in their file names and calculating the overlap of the images, no further computing was needed and the images were just placed slightly overlapping to each other. The overlap was approximately 20% of the whole length of the images. To perform the stitching automatically, the stitching plugin for grid/collection stitching in *Fiji* was employed.

### 2.3 Variance analysis

In order to reduce the dimensionality of the 3D data cube to 2D, variance images were created. The variance for every single pixel of the images in the observed spectral range (the 3D datacube) was calculated. This quantity shows how much the brightness is changing over the acquired spectral region. The variance enhances strong differences in the data more than the SD would, in this way leading to reduced influence of statistical artifacts:

ImgVarx,y=1n∑Imgx,y−Imgx,y,λ2.

Img_Var_ corresponds to the resulting variance image. *x*, *y* corresponds to the pixel positions. *λ* corresponds to the wavelength. Img(*x*, *y*, *λ*) is the acquired image at the wavelength lambda and Img(*x*, *y*) corresponds to the average image (pixel values) along the lambda dimension. The idea behind this approach is to summarize all the differences over the spectral range: stronger differences should result from higher concentrations of absorbing substances in the tissues and this will consequently result in different variances for different tissues. Statistical tests were performed using the statistics toolbox in Microsoft Excel (*t* test and *f* test).

### 2.4 Sample preparation

To assess the feasibility of analyzing histological samples for the diagnosis of skin cancer, fixed and paraffin‐embedded samples of patients with a clear histological diagnosis were used. Use of skin samples was approved by the Ethics Committee of the University Medicine Göttingen (UMG, protocol number: 3/1/19). Fixation, embedding, sectioning and staining (if applicable) were carried out according to standard procedures.

*Fixation*: Freshly excised skin samples were fixed in 4% formalin in NaCl solution for at least 2 hours (up to 24 hours). As a result of this fixation method, some proteins are denatured. Lipids and associative carbohydrates are not fixated.

*Embedding*: Most samples are embedded in paraffin; therefore, the sample is soaked in liquid paraffin. Since this molecule does not mix with water, the sample has to be first dehydrated. This step is achieved by repeated washing of the sample with a mixture of ethanol and water, where the ethanol/water ratio shifts with every step towards pure ethanol. By doing that, most lipids are removed from the sample. After embedding in the liquid paraffin, the paraffin block is left to harden in order to form a block that can be cut using a microtome.

*Cutting*: Samples were placed upon the microtome (RM 2235, Leica) and cut at 3‐μm thick sections. Sections were then placed on a glass slide (J1800AMNZ, Thermo Scientific).

*Staining*: For the application of the staining, the sections were deparafinized and rehydrated first in xylene then in decreasing concentrations of ethanol. Sections were then stained using the two dyes, eosin (Shandon Eosin Y Aqueous, Thermo Scientific) and HE (Mayer Hämatoxylin, Q Path) in an automated staining machine (Tissue Tek Prisma, Sakura).

At the end, samples were mounted using mounting medium Cytoseal 60 (Thermo Scientific) and coverslips (MEZ102460, Thermo Scientific). In case of nonstained sampled, the procedure was stopped after deparaffinization and rehydration and slides were mounted in the same way as described above.

## 3 RESULTS AND DISCUSSION

**Figure 2:**
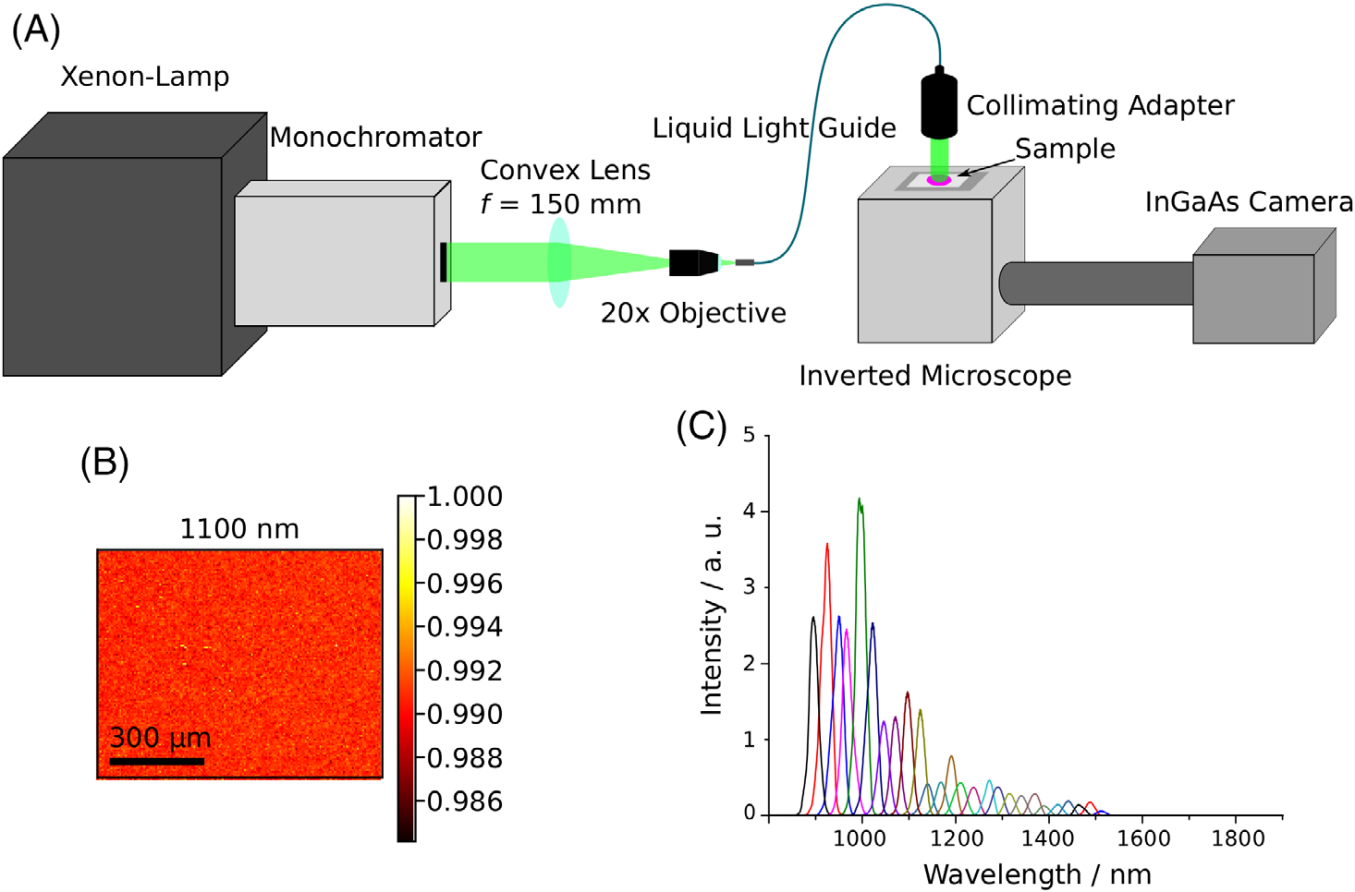
Schematic diagram of the multispectral NIR absorption setup. A, General overview of the setup consisting of a broadband light source, a monochromator, optics to enable wide‐field transmission illumination, an inverted microscope and a cooled NIR InGaAs camera. B, Image acquired with the monochromator setup on a blank sample, intensity normalized to the highest intensity in the image. The high homogeneity of the intensity distribution in the image is clearly observable. C, Spectra of the monochromator lamp. The FWHM of the single Gaussians are about 25 nm and the setup covered potentially the spectral range from 400 nm up to 1500 nm. For this experiment, the range between 900 and 1500 nm only was used

To perform NIR multispectral imaging, an optical setup was built around a standard inverted microscope (Figure 2A). The light source for illuminating the sample was a xenon lamp that released a broad range of wavelengths. Single wavelengths were selected by a monochromator. The light beam released by the monochromator was focused with a lens (*f* = 150 mm) and a ×20 objective into a liquid light guide and then into a collimating adapter lens. This lens was placed above the sample (10 cm) to enable homogenous illumination of the sample similar to a standard bright field microscope setup. A homogeneously illuminated NIR image is shown in Figure 2B.

None of the optical elements had any antireflective NIR coating to guarantee high transmission efficiencies in the NIR. With this setup, it was possible to select wavelengths with a *full width at half maximum (FWHM)* of 25 nm (Figure 2C). This monochromator‐based setup was used to analyze skin samples. Additionally, a multispectral imaging setup based on a broad band NIR light source and bandpass filters to select single spectral regions was built (Figure S1). It does not require a monochromator and a special illumination path but the spectral resolution is limited by the number of filters (up to 6 in our case).

For this study, HE‐stained and non‐HE‐stained fixed samples of melanoma, squamous‐cell carcinoma and nodular basal‐cell carcinoma were used. The samples had been previously diagnosed by an experienced histologist to compare and benchmark our results. In order to ensure that the HE dyes did not influence the NIR measurements, absorption spectra of the dyes were measured.

In general, the HE dyes showed nearly no absorption in the NIR (Figure S2). In comparison to a standard NIR absorbing material such as single‐walled carbon nanotubes the background absorption appeared to be negligible 15.

**Figure 3:**
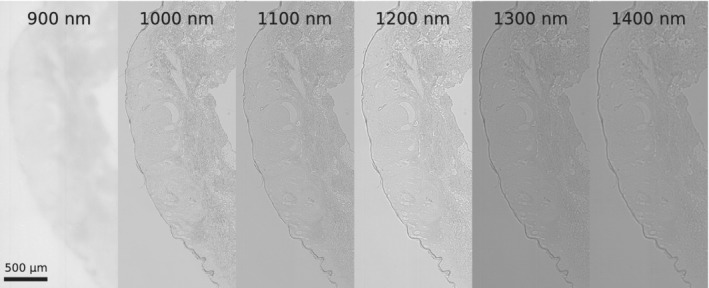
Images of skin samples at different wavelengths. A HE‐stained sample of the nodular basalioma was measured in the NIR at the indicated wavelengths. The optical setup was optimized for wavelengths >900 nm and therefore the 900 nm appears to be less sharp

**Figure 4:**
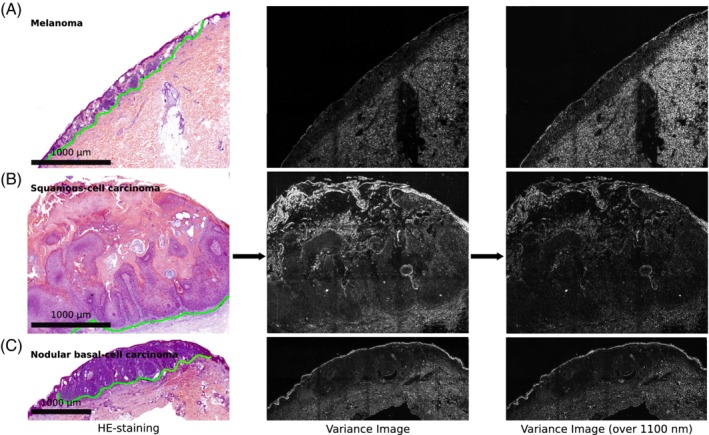
Near infrared (NIR) spectral variance images of different skin diseases. On the left side different HE‐stained skin samples are shown. Of these samples NIR absorption images were collected at different wavelengths. Here, the variance images (along the spectral dimension) for the full spectral range (30 wavelengths) and the >1100 nm range (20 wavelengths) are shown. The green dotted line in the HE staining indicates the border between normal tissue and tumor. A, Melanoma; B, Squamous‐cell carcinoma and C, Nodular basal‐cell carcinoma. The scale bars in the bright field apply also for the corresponding variance images. The tumor area is located above the green line in each sample

**Figure 5:**
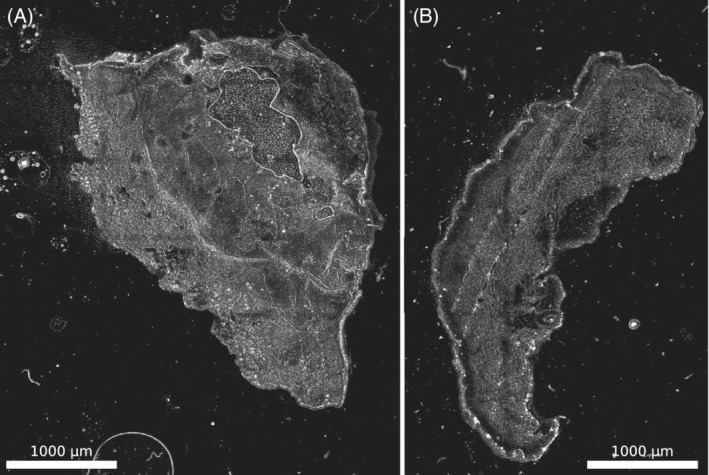
Near infrared (NIR) variance images of unstained samples. Here, the variance along the full spectrum was calculated for nonstained samples (from the same tissue samples as in Figure 4). A, Squamous‐cell carcinoma; B, nodular basal‐cell carcinoma

**Figure 6:**
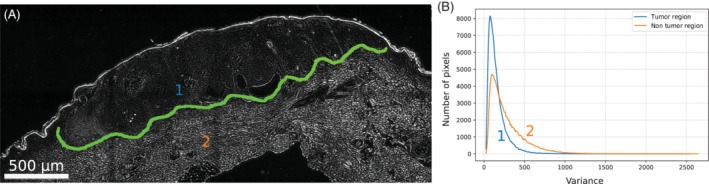
Near infrared (NIR) variance image and quantification of tissue differences. A, Variance image of the nodular basal‐cell carcinoma. The blue and the orange number indicate the region, where the histograms shown in B were measured. B, Histograms of the variance values in the tumor and nontumor regions. Both shape and average variance differ in the two regions. The two distributions are statistically different (*f* test, *P* < .001)

Nevertheless, there was a small absorption shoulder at around 1000 nm and we could not rule out that in a skin sample the local concentration of the HE dyes would be larger. Therefore, we always collected images at all different NIR wavelengths and analyzed either all wavelengths or the wavelengths >1100 nm that had unambiguously no HE absorption feature. The results (Figures 3, 4, 5, 6) show that this HE absorption feature is negligible. In the next step, different skin samples were imaged using the above‐described setup.

For multispectral images, large parts of the sample including tumor and nontumor area were imaged. Raw NIR images are shown in Figure 3 and reveal the NIR contrast. At a wavelength of 900 nm images appeared less sharp, which we attributed to lower quantum efficiencies of the InGaAs camera (in this range) and lower penetration depths at this wavelength.

Multispectral images I(*x*, *y*, *λ*) represent 3D data cubes with a wavelength axis and *x*, *y* pixel positions. Typically, images at 30 different wavelengths (920‐1500 nm, 20 nm steps) were acquired. Such data sets could be analyzed with different approaches from image analysis. As a first step we decided to perform a variance analysis along the wavelengths axis as described in the methods part. The results of this variance analysis were compared to visible HE images (Figure 4). The variance in the nontumor tissue is significantly higher (corresponding to higher brightness) compared to the variance in the tumor tissue (both for all wavelengths and only wavelengths >1100 nm).

To show that the contrast is indeed due to differences in NIR absorption we also used unstained slices from the samples shown in Figure 4. It shows a similar contrast pattern (Figure 5), which highlights that our approach will work with nonstained samples, which would be a clear advantage compared to HE staining protocols. Additionally, the bandpass filter setup mentioned before yielded similar results and images (Figures S3 and S4).

Next, differences between the tumor areas (defined by a histologist using the HE images) were quantified by comparing the variance (squares of 45 × 45 pixels) from tumor and nontumor regions. A similar analysis has been performed in the past to quantify the increase in contrast 27.

As a measure of our method, the ratios of these variance values of the tumor vs the nontumor regions were used (Table 1). These ratios would be 1 if there is no difference. As a comparison the contrast from the raw data at 1300 nm is provided, which shows that the spectral domain significantly improves the sensitivity of this approach. Other wavelengths showed similar raw contrast values. The ratios between tumor and nontumor tissue were 0.45 ± 0.05 (nodular *basal‐cell carcinoma*), 0.58 ± 0.07 (*squamous‐cell carcinoma*) and 0.40 ± 0.06 (*melanoma*). The error corresponds to the SD (n = 4 different regions of a sample from one patient). These values suggest that it is possible to distinguish in all three cases tumor from nontumor tissue. This finding is illustrated by histograms of the variance image pixel intensities of a tumor vs a nontumor region (Figure 6).

**Table 1:** Ratio of the mean variances of the tumor area and the nontumor area for different samples (normalized to the nontumor area, errors are SD, n = 4 regions from one tumor sample)

Diagnosis	Ratio of variances	Ratio of variances (>1100 nm)	Raw data (1300 nm)
Melanoma	0.45 ± 0.05	0.38 ± 0.07	0.998 ± 0.002
Nodular basal‐cell carcinoma	0.58 ± 0.07	0.55 ± 0.09	0.998 ± 0.002
Squamous‐cell carcinoma	0.40 ± 0.06	0.36 ± 0.09	1.009 ± 0.002

Additionally, nodular basal‐cell carcinoma samples from three different patients were analyzed in the same way (Table 2). The variance values vary between different patients most likely because of differences in the sample preparation such as sample thickness etc. However, the ratios of variance values of tumor vs nontumor region were very consistent. Without normalization a statistical test (paired *t* test) yielded *P* < .14 and *P* < .095 (>1100 nm). With normalization to the absolute values of the nontumor tissue yielded *P* < .01 (both whole wavelength range and >1100 nm). These results indicate that NIR multispectral imaging provides significant information about this type of cancer and is consistent for samples from different patients. Additionally, the results show that it is not that the one wavelength alone that provides the necessary information but the whole spectral range.

**Table 2:** Near infrared (NIR) variance differences for nodular basal‐cell carcinoma samples from three different patients

Patient	Variance tumor area	Variance nontumor area	Variance tumor area (>1100 nm)	Variance nontumor area (>1100 nm)	Ratio (tumor/nontumor)	Ratio (tumor/nontumor) (>1100 nm)
1	146	274	57	117	**0.53**	**0.49**
2	54	98	24	44	**0.55**	**0.55**
3	94	159	32	73	**0.59**	**0.44**

Absolute values vary but the ratios between tumor/nontumor regions are consistent.

## 4 CONCLUSION

Our results show how to setup a multispectral NIR absorption imaging setup and use it for histology of skin cancer samples. In comparison to other methods our approach can be easily implemented into an existing microscope setup 4, 28. Multispectral imaging results in large amounts of data and we show that even the simplest approach to use the spectral information (variance images) enabled us to distinguish tumor from nontumor regions. In the future, more sophisticated image analysis methods and concepts from artificial intelligence could be used to better interpret such data 29, 30, 31, 32.

The data presented in this work is a proof of principle that multispectral NIR imaging could become an important tool for histology and diagnostics. The samples used in this study were fixed samples that underwent a solvent extraction process, which removes lipids and other substances that would further enhance the NIR contrast. Therefore, we anticipate that nonstained, nontreated samples or in vivo experiments would show even more differences and better NIR contrast.

There are already more established techniques such as OCT, which is based on differences in refractive index 33. Another technique is Raman imaging, which provides rich chemical information but is experimentally more difficult to implement 4, 34, 35, 36. The approach presented in this work combines certain advantages of those two methods and could be extended to in vivo diagnostics. In summary, multispectral NIR imaging combines the advantages of deep tissue penetration by NIR light with chemical information from spectral information and could become a new powerful tool in skin histology.

## Supporting information


**Figure S1**. Bandpass filter setup. A, Design of the bandpass filter setup. B, The intensity results from the average intensity of a single pixel in an image recorded with the InGaAs Camera.
**Figure S2**. Absorption of HE dyes in the NIR. Absorption spectra of the HE dyes in comparison with a sample of (6,5)‐carbon nanotubes with a concentration of around 1 mg/L.
**Figure S3**. Raw data from the bandpass filter setup.
**Figure S4**. Variance analysis of the data from the bandpass filter setup compared to the brightfield images.
**Figure S5**. Variance analysis of additional nodular basal‐cell carcinoma from different patients compared to HE images.
**Appendix S1**. Bandpass filter setup.
