# Supplementary material for: Multispectral near infrared absorption imaging for histology of skin cancer
Source: J Biophotonics. 2019 Nov 5;13(1):e201960080. doi: 10.1002/jbio.201960080 (PMC7065629; doi:10.1002/jbio.201960080)
Supplement: Supplementary file 1 — Figure S1. Bandpass filter setup. A, Design of the bandpass filter setup. B, The intensity results from the average intensity of a single pixel in an image recorded with the InGaAs Camera. Figure S2. Absorption of HE dyes in the NIR. Absorption spectra of the HE dyes in comparison with a sample of (6,5)‐carbon nanotubes with a concentration of around 1 mg/L. Figure S3. Raw data from the bandpass filter setup. Figure S4. Variance analysis of the data from the bandpass filter setup compared to the brightfield images. Figure S5. Variance analysis of additional nodular basal‐cell carcinoma from different patients compared to HE images. Appendix S1. Bandpass filter setup. [file JBIO-13-e201960080-s001.pdf]

Supplementary Information

Multispectral NIR absorption imaging for histology of skin cancer

Alexander Spreinat[1], Gabriele Selvaggio[1], Luise Erpenbeck[2], Sebastian Kruss[1*]*

[1]Institute of Physical Chemistry, Göttingen University, Germany

[2] Department of Dermatology, Venereology and Allergology, University Medical Center, Göttingen, Germany

*corresponding author: skruss@gwdg.de (Sebastian Kruss), luise.erpenbeck@med.uni-goettingen.de

Bandpass Filter Setup

A simple approach to perform multispectral imaging is to use bandpass filters to select spectral bands for the multispectral image. Before we implemented a monochromator setup we established a straightforward bandpass filter setup (figure S1a). The sample was illuminated from above with a built-in microscope lamp (U-LH100L-3). This lamp was a broad band light source with a significant emission in the NIR range. The intensity of the signal in each spectral range was measured and quantified (figure S1b). After the light has passed through the sample and was partially absorbed, a filter wheel was placed in the light path and equipped with 5 different bandpass filters (980 nm, 1100 nm, 1200 nm, 1300 nm, 1400 nm). By doing that, a single spectral region was selected before the light fell onto the InGaAs Camera. The data that was generated by this setup is shown in the supplement information in figure S3 and S4. The setup itself is shown in figure S1.

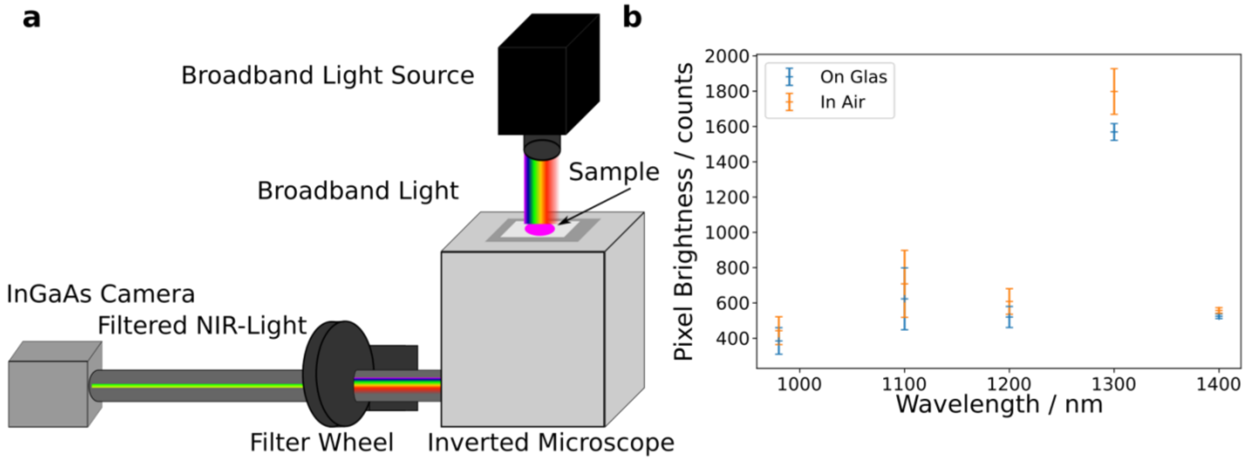

Figure S1: Bandpass filter setup. a: Design of the bandpass filter setup. **b**: The intensity results from the average intensity of a single pixel in an image recorded with the InGaAs Camera. The error bars correspond to the standard deviation of all pixels (n=320x256).

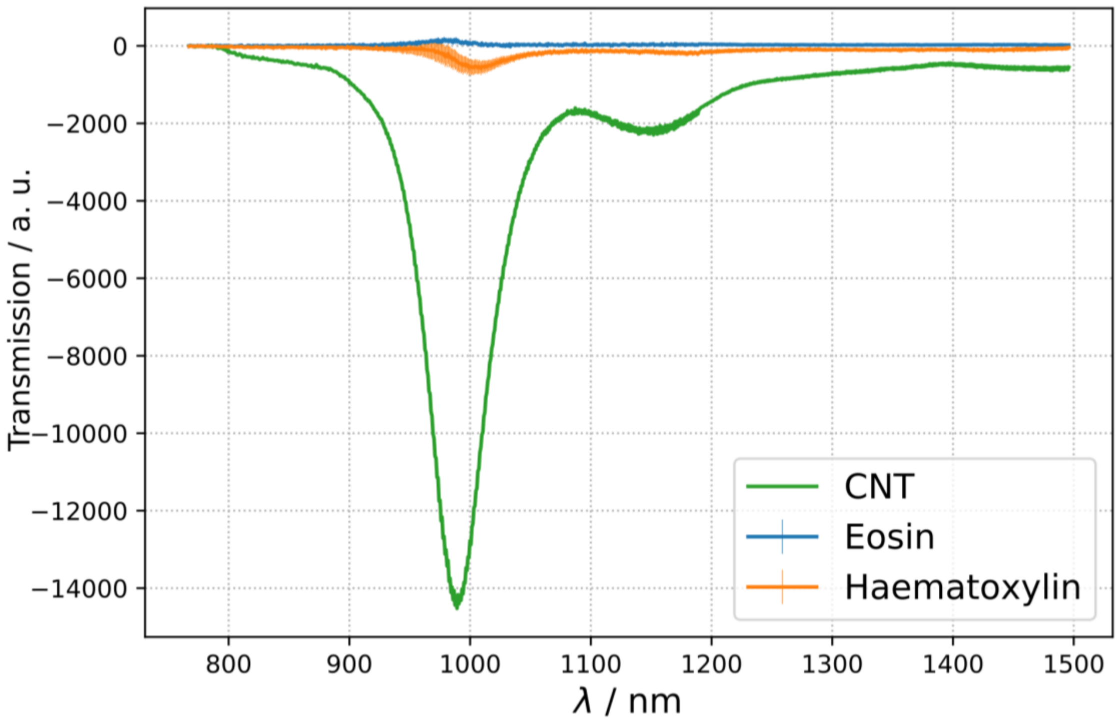

Figure S2: Absorption of HE dyes in the NIR. Absorption spectra of the HE dyes in comparison with a sample of (6,5)-carbon nanotubes with a concentration of around 1 mg/l. The concentration of the dyes was approximately 3 g/l. For this measurement, a drop of each dye and the carbon nanotubes was placed on a glass slide and dried. The dyes show a weak absorption around 1000 nm. Even though it is very weak it could affect nIR images of HE stained samples. That leads to the conclusion that longer wavelengths should not be influenced by the dyes.

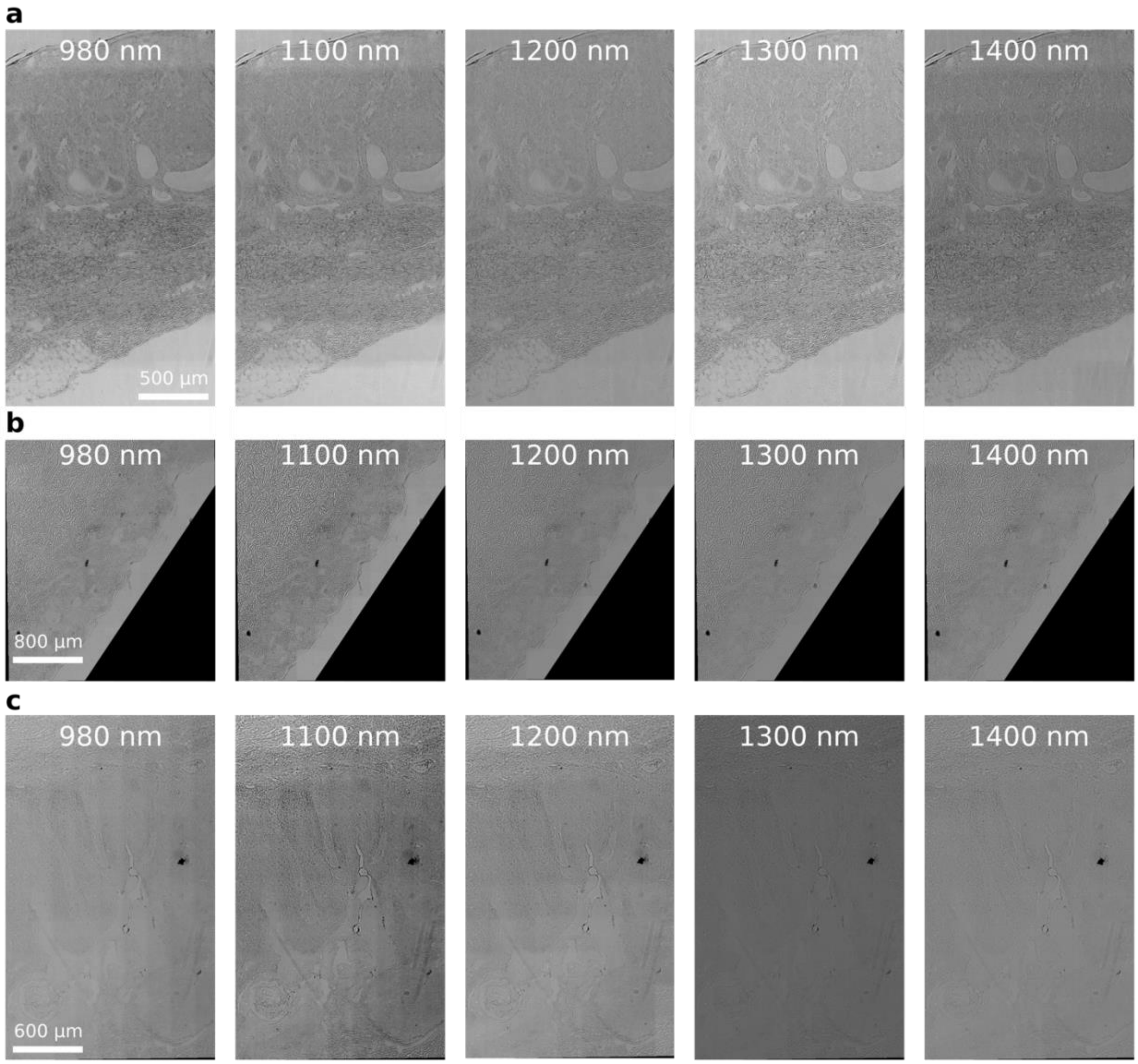

Figure S3: Raw data from the bandpass filter setup. a Nodular basal-cell carcinoma **b** Melanoma **c** Squamous-cell carcinoma sample. Bright field images of the samples can be found in S4.

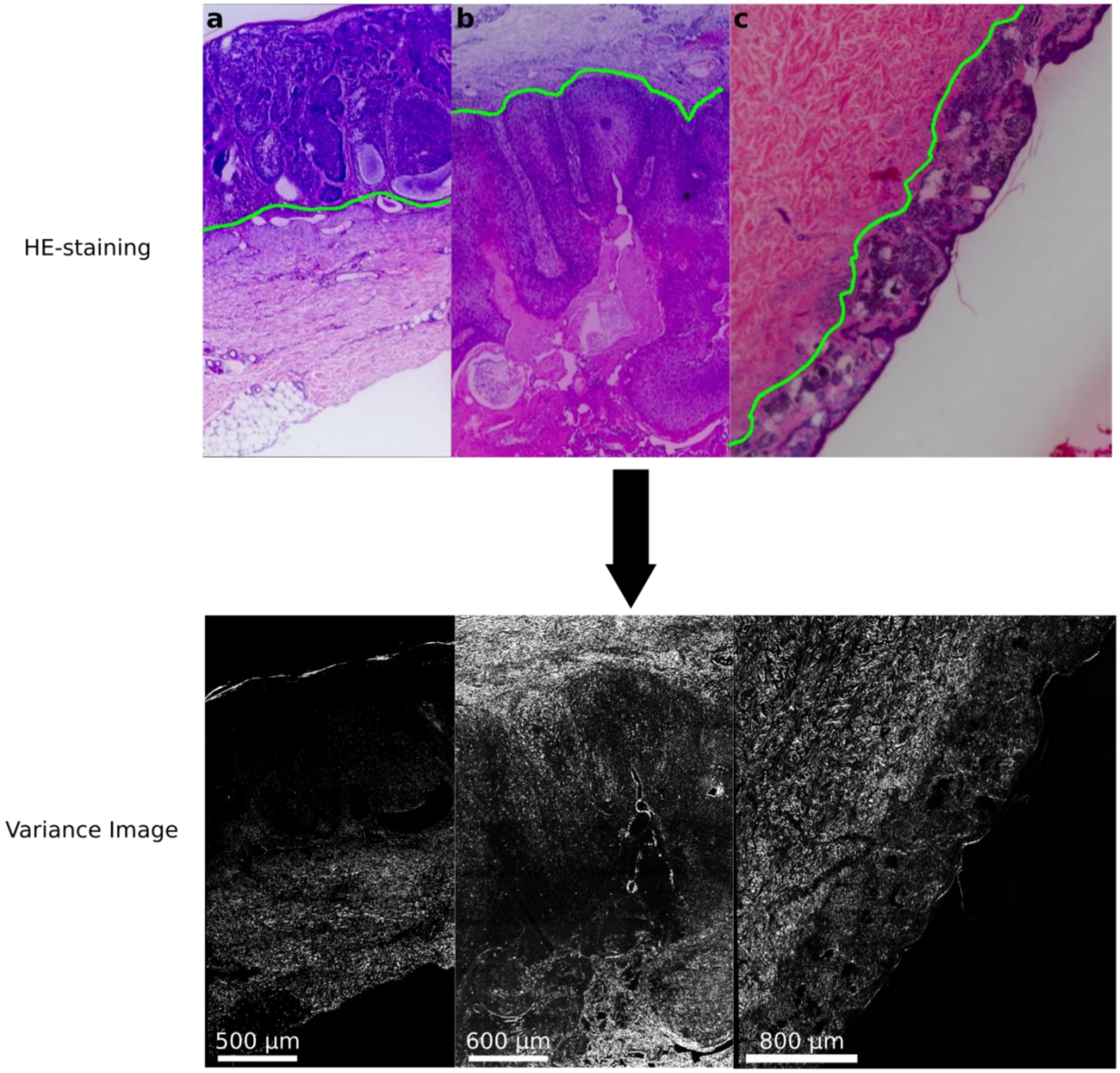

Figure S4: Variance analysis of the data from the bandpass filter setup compared to the brightfield images. a Nodular basal-cell carcinoma, tumor above the green line, **b** Squamous-cell carcinoma, tumor below the green line, **c** Melanoma, tumor below the green line.

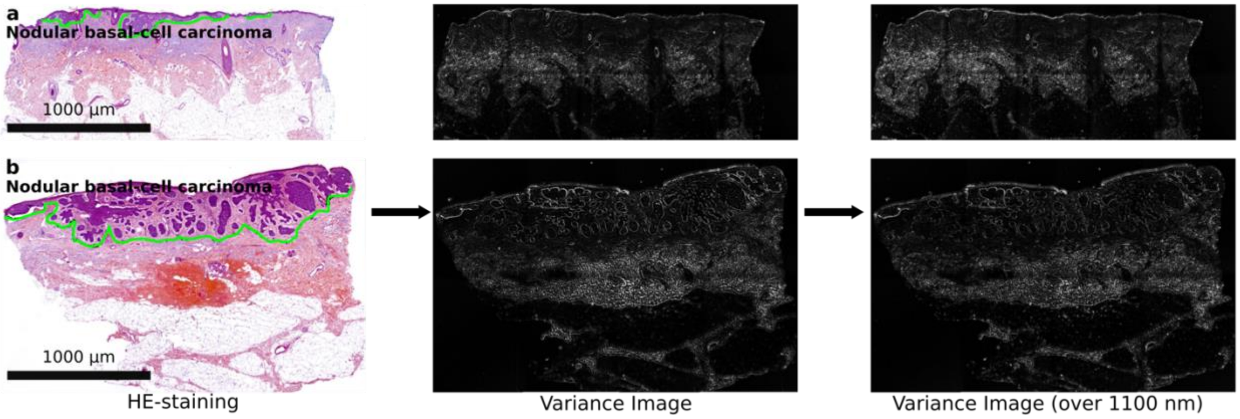

Figure S5: Variance analysis of additional nodular basal-cell carcinoma from different patients compared to HE images. a Patient 2 **b** Patient 3. Tumor above the green line for both samples
